# ﻿A new species of karst-adapted gecko (Squamata, Gekkonidae, *Gekko*) from Guangxi, southern China

**DOI:** 10.3897/zookeys.1245.153769

**Published:** 2025-07-15

**Authors:** Zhong Huang, Hao-Tian Wang, Shuo Qi, Han-Ming Song, Yong Huang, Ying-Yong Wang, Yun-Ming Mo

**Affiliations:** 1 Natural History Museum of Guangxi, Nanning 530012, China Natural History Museum of Guangxi Nanning China; 2 School of Ecology / School of Life Sciences, Sun Yat-sen University, Guangzhou 510275, China Sun Yat-sen University Guangzhou China; 3 Guangxi University of Chinese Medicine, Nanning 530200, China Guangxi University of Chinese Medicine Nanning China

**Keywords:** *Gekkofengshanensis* sp. nov., *G.liboensis*, Guangxi, integrative taxonomy, *Japonigekko*, South China Karst

## Abstract

A new species of the genus *Gekko* Laurenti, 1768, *Gekkofengshanensis***sp. nov.**, is described based on six specimens from Fengshan County, Hechi City, Guangxi Zhuang Autonomous Region, China. It is placed into the subgenus Japonigekko based on morphological and molecular phylogenetic analyses, and distinguished from consubgeners of *Japonigekko* by a combination of morphological characters in body size, cephalic proportions, and pholidosis features. Molecular phylogenetic analyses using mitochondrial 16S and ND2 sequences reveal that *G.fengshanensis* sp. nov. forms a sister relationship with *G.kwangsiensis*, collectively forming a clade with *G.liboensis* and *G.paucituberculatus* endemic to southern China’s karst ecosystems. This discovery increases the number of recognized *Gekko* species in the South China Karst to five, underscoring the role that fragmented karst landscapes play in driving speciation and maintaining high levels of biodiversity in this unique ecosystem.

## ﻿Introduction

The genus *Gekko* Laurenti, 1768, currently containing 92 known species, is a widely distributed group of nocturnal gekkonid lizards, mainly distributing in throughout plains and plateaus across temperate and tropical Asia and the western islands of Pacific Ocean (Cao et al. 2025; Pauwels et al. 2025; Uetz et al. 2025). According to recent phylogenetic studies based on genomic data, the genus is currently divided into seven subgenera, namely *Archipelagekko*, *Balawangekko*, *Gekko*, *Japonigekko*, *Lomatodactylus*, *Ptychozoon*, and *Rhacogekko* (Wood et al. 2020). Among these subgenera, *Japonigekko* exhibits the highest diversity and contains the greatest number of species in China. Its distribution spans across the country, from Liaoning in the northeast to Xizang in the west, and down to Hainan in the south, thriving in a variety of habitats (Qiu et al. 2023; Ma et al. 2024). Notably, Guangxi is home to the largest number of species within this subgenus.

Guangxi’s karst topography, exemplified by iconic formations such as the Leye Giant Sinkhole Cluster and the Guilin Tower Karst, forms an integral component of the South China Karst, a UNESCO World Heritage Site. This lithological mosaic has facilitated the radiation of specialized herpetofauna, with recent decades witnessing the discovery of multiple karst-obligate species including the frog *Odorranalipuensis* Mo, Chen, Wu, Zhang & Zhou, 2015, the geckos *Gekkokwangsiensis* Yang, 2015 and *G.paucituberculatus* Wang, Qi, Zhou & Wang, 2024, and other saxicolous taxa. In 2024, during two field surveys in the karst forests of northwest Guangxi, six *Gekko* individuals were collected. Morphologically, these specimens clearly belong to subgenus Japonigekko, characterized by relatively moderate size; nares in contact with rostral; the presence of dorsal tubercle rows and precloacal pores; and lacking tubercles on ventrolateral folds. Phylogenetic analysis places them in a distinct evolutionary lineage, separate from closely related congeners within the subgenus. This suggests they represent a previously undescribed species, distinguished by both molecular and morphological differences. In light of these findings, we provide a detailed description of the new species herein.

## ﻿Materials and methods

### ﻿Specimens and morphology

**Figure 1.**
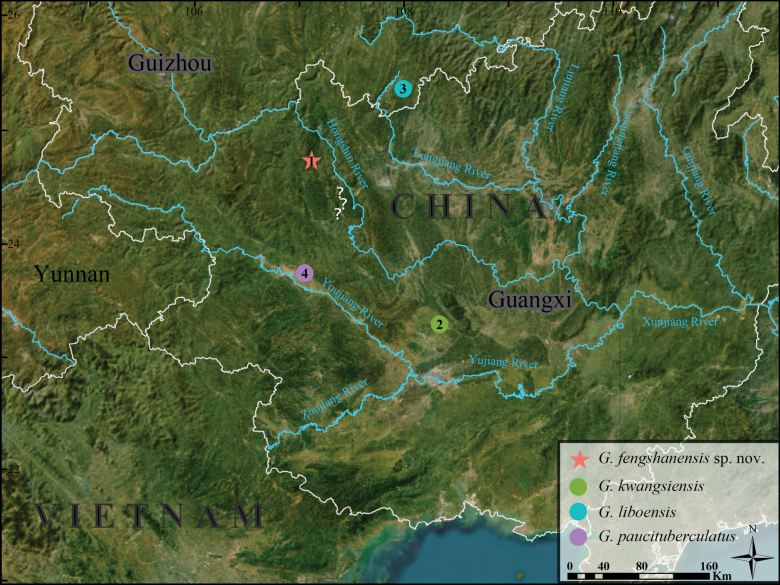
Localities of *Gekkofengshanensis* sp. nov., *G.kwangsiensis*, *G.liboensis*, and *G.paucituberculatus*. 1 Fengcheng Town, Fengshan County, Hechi City, Guangxi; 2 Wuming District, Nanning City, Guangxi; 3 Maolan National Nature Reserve, Libo County, Guizhou Province; 4 Tianyang District, Baise City, Guangxi. “?” indicates the unconfirmed locality of *G.fengshanensis* sp. nov.

Six specimens of *Gekkofengshanensis* sp. nov. were collected from Fengshan County, Hechi City, Guangxi Zhuang Autonomous Region, China in 2024 (Fig. 1). The specimens were euthanized and then fixed in 10% buffered formalin, later transferred to 75% ethanol and deposited in the Natural History Museum of Guangxi (NHMG), Nanning and Sun Yat-sen University (SYS), Guangzhou, China. Liver tissue samples were preserved in 95% ethanol for molecular analysis of four specimens (NHMG 240713/SYS r002885, NHMG 240714/SYS r002886, NHMG 202408005, 006). Two specimens of *G.liboensis* (SYS r002876, 77) were also collected and sequenced.

Measurements were taken with digital calipers (Deli DL91200 Digital Vernier Caliper) to the nearest 0.1 mm on the right side of the body, and scalation features were counted under a binocular scope (Leica EZ4 HD). Bilateral scale counts are given as left/right. External measurements, meristic traits and their abbreviations followed Wang et al. (2024). They are snout-vent length (**SVL**, from tip of snout to anterior margin of cloaca) ; tail length (**TaL**, from posterior margin of cloaca to tip of tail) ; axillia-groin length (**>AG**, distance between axilla and groin) ; head length (**HL**, from tip of snout to posterior margin of ear opening) ; head width (**HW**, at the angle of the jaws) ; head height (**HH**, from the top of the head posterior to the eyes to the bottom of the lower jaw) ; snout length (**SNT**, from snout tip to anterior corner of eye) ; maximum eye diameter (**ED**) ; maximum ear opening diameter (**EOD**) ; maximum rostral width (**RW**) ; maximum rostral height (**RH**) ; maximum mental length (**ML**). Meristic characters are nasals (**N**, nasorostrals, supranasals and postnasals) ; intersupranasals (**I**, scales between supranasals, in contact with rostral ; supralabials and infralabials (**SPL and IFL**, number of scales from commissure of jaw to the rostral /mental scale) ; interorbitals (**IO**, number of scales in a line between anterior margins of eyes) ; preorbitals (**PO**, number of scales in a line from nostril to anterior margin of the eye) ; postmentals (**PM**, scales bordering the mental) ; gulars bordering the postmentals (**GP**) ; dorsal tubercle rows at midbody (**DTR**) ; granules surrounding dorsal tubercles (**GSDT**) ; scales in a line from mental to the front of cloacal slit (**SMC**) ; ventral scale rows at midbody between ventrolateral folds (**V**) ; scale rows at midbody (**SR**, including ventral scales) ; subdigital lamellae of entire first finger (**LF1**) ; subdigital lamellae of entire fourth finger (**LF4**) ; subdigital lamellae of entire first toe (**LT1** ; subdigital lamellae of entire fourth toe (**LT4**) ; precloacal pores (**PP**) ; postcloacal tubercles (**PAT**) ; transverse dorsal scale rows in the middle of the third caudal whorl (**S3W**). The information on character states of other *Japonigekko* species was obtained from the literature (Table 1).

**Table 1.** Literature and authorities for species of *Japonigekko* morphological characters used in this study.

ID	Species	References
1	*Gekkoaaronbaueri* Ngo, Thai, Phimvohan, David & Teynié, 2015	Ngo et al. (2015)
2	*G.adleri* Nguyen, Wang, Yang, Lehmann, Le, Ziegler & Bonkowski, 2013	Nguyen et al. (2013)
3	*G.alpinus* Ma, Shi, Shen, Chang & Jiang, 2024	Ma et al. (2024)
4	*G.auriverrucosus* Zhou & Liu, 1982	Zhou et al. (1982)
5	*G.bonkowskii* Luu, Calame, Nguyen, Le & Ziegler, 2015	Luu et al. (2015)
6	*G.canhi* Rösler, Nguyen, Van Doan, Ho, Nguyen & Ziegler, 2010	Rösler et al. (2010)
7	*G.chinensis* (Gray, 1842)	Ota et al. (1995)
8	*G.cib* Lyu, Lin, Ren, Jiang, Zhang, Qi & Wang, 2021	Lyu et al. (2021)
9	*G.guishanicus* Lin & Yao, 2016	Lin and Yao (2016)
10	*G.hokouensis* Pope, 1928	Zhou et al. (1982)
11	*G.ichangensis* Cao, Sucharitakul, Tie, Suwannapoom, Yan & Chomdej, 2025	Cao et al. (2025)
12	*G.japonicus* (Schlegel, 1836)	Zhou et al. (1982)
13	*G.jinjiangensis* Hou, Shi, Wang, Shu, Zheng, Qi, Liu, Jiang & Xie, 2021	Hou et al. (2021)
14	*G.kaiyai* Zhang, Wu & Zhang, 2023	Zhang et al. (2023)
15	*G.khunkhamensis* Sitthivong, Lo, Nguyen, Ngo, Khotpathoom, Le, Ziegler & Luu, 2021	Sitthivong et al. (2021)
16	*G.kwangsiensis* Yang, 2015	Yang (2015)
17	*G.liboensis* Zhou, Liu & Li, 1982	This study
18	*G.melli* (Vogt, 1922)	Lyu et al. (2021)
19	*G.nadenensis* Luu, Nguyen, Le, Bonkowski & Ziegler, 2017	Luu et al. (2017)
20	*G.palmatus* Boulenger, 1907	Ota et al. (1995); Song et al. (2024)
21	*G.paucituberculatus* Wang, Qi, Zhou & Wang, 2024	Wang et al. (2024)
22	*G.scabridus* Liu & Zhou, 1982	Zhou et al. (1982)
23	*G.scientiadventura* Rösler, Ziegler, Vu, Herrmann & Böhme, 2004	Rösler et al. (2004)
24	*G.sengchanthavongi* Luu, Calame, Nguyen, Le & Ziegler, 2015	Luu et al. (2015)
25	*G.shibatai* Toda, Sengoku, Hikida & Ota, 2008	Toda et al. (2008)
26	*G.similignum* Smith, 1923	Ota et al. (1995)
27	*G.subpalmatus* (Günther, 1864)	Lyu et al. (2021)
28	*G.swinhonis* Günther, 1864	Rösler et al. (2011)
29	*G.taibaiensis* Song, 1985	Song (1985)
30	*G.tawaensis* Okada, 1956	Rösler et al. (2011)
31	*G.thakhekensis* Luu, Calame, Nguyen, Le, Bonkowski & Ziegler, 2014	Luu et al. (2014)
32	*G.truongi* Phung & Ziegler, 2011	Phung and Ziegler (2011)
33	*G.vertebralis* Toda, Sengoku, Hikida & Ota, 2008	Toda et al. (2008)
34	*G.vietnamensis* Sang, 2010	Sang (2010)
35	*G.wenxianensis* Zhou & Wang, 2008	Zhou and Wang (2008)
36	*G.yakuensis* Matsui & Okada, 1968	Rösler et al. (2011)

The morphometric measurements were statistically analyzed using R v. 4.4.2 (R Core Team, 2024). For analyses, all measurements were ln-transformed to normalize and reduce the variance, and then scaled to remove allometric effects of body size using the following equation: X_a_ = X_ln_ - *β* · (SVL_ln_ - SVL_m_), where X_a_ = adjusted value; X_ln_ = ln-transformed measurements; *β* = unstandardized regression coefficient for each species; SVL_ln_ = ln-transformed SVL; and SVL_m_ = overall average SVL_ln_ of each species. One-way analysis of variance (ANOVA) was conducted with statistically similar variances (*p* > 0.05 in the Levene’s test) and performed on a dataset coded for species to examine statistically significant mean differences (*p* < 0.05) among characters using the *car* R package. Character means showing significant differences were subjected to a Tukey HSD test to determine which pairs of species differed significantly for those specific characters. Principal component analysis (PCA) was performed to cluster the morphometrics except SVL related to each species using *GroupStruct* R package (Chan and Grismer 2022). Multiple Factor Analysis (MFA) is a multivariate approach for integrating multiple sets of quantitative traits into a single analysis (Pagès 2015). Each dataset is first analyzed separately using PCA and then standardized by dividing all values by the square root of the first eigenvalue. The standardized datasets are then combined for a global PCA. This ensures that all trait sets contribute equally to the overall variation (Grismer et al. 2024).

To evaluate morphological differences among species or populations, we performed a non-parametric permutation multivariate analysis of variance (PERMANOVA) from the *vegan* package in R (Oksanen et al. 2020) on the PCA scores and the first five MFA dimensions. This test, based on Euclidean distances and 50,000 permutations, assesses whether the group centroids and dispersions differ significantly in multivariate space. A significant adjusted *p*-value (< 0.05) indicates clear separation among groups. Given the number of individuals available for closely related species, we performed ANOVA comparisons only with *Gekkokwangsiensis*, while in the PCA and MFA, we include all three (*G.kwangsiensis*, *G.liboensis*, and *G.paucituberculatus*) for visualization. The raw data are given in Suppl. material 1.

### ﻿Phylogenetic sampling and analyses

Twelve new sequences and 51 sequences from GenBank were used for molecular analysis in this study. All newly collected tissue samples were obtained from euthanized specimens and then preserved in 95% ethanol and stored at -40 °C. *Gekkogecko* (Linnaeus, 1758) and *G.reevesii* (Gray, 1831) belonging to subgenus Japonigekko were used to root the tree based on Rösler et al. (2011) and Lyu et al. (2021). Detail information of these samples is given in Table 2.

**Table 2.** Localities, voucher information, and GenBank accession numbers for all samples used in this study.

ID	Species	Locality	Voucher ID	16S	ND2	Reference
**1**	***Gekkofengshanensis* sp. nov.**	**China: Guangxi: Hechi: Fengshan**	**NHMG 240713/SYS r002885**	**PV652773**	**PV657377**	**This study**
**2**	***Gekkofengshanensis* sp. nov.**	**China: Guangxi: Hechi: Fengshan**	**NHMG 240714/SYS r002886**	**PV652774**	**PV657378**	**This study**
**3**	***Gekkofengshanensis* sp. nov.**	**China: Guangxi: Hechi: Fengshan**	**NHMG 202408005**	**PV652775**	**PV657379**	**This study**
**4**	***Gekkofengshanensis* sp. nov.**	**China: Guangxi: Hechi: Fengshan**	**NHMG 202408006**	**PV652776**	**PV657380**	**This study**
5	*G.adleri*	China: Guangxi: Jingxi	SYS r001400	MW451654	OR902178	Lyu et al. (2021); Wang et al. (2024)
6	*G.alpinus*	China: Xizang: Mangkang	CIB 121656	PQ255976	PQ303494	Ma et al. (2024)
7	*G.auriverrucosus*	China: Shanxi: Yuncheng	NNU Z 20050716.004	—	JN019062	Rösler et al. (2011)
8	*G.bonkowskii*	Laos: Khammouane	VFU R.2014.10	—	KT266818	Luu et al. (2015)
9	*G.chinensis*	China: Hong Kong	SYS r001211	MW451644	OR902183	Lyu et al. (2021); Wang et al. (2024)
10	*G.cib*	China: Sichuan: Hejiang	SYS r001489	MW451655	OR902165	Lyu et al. (2021); Wang et al. (2024)
11	*G.hokouensis*	China: Jiangxi: Mt. Meiling	SYS r001311	MW451648	OR902172	Lyu et al. (2021); Wang et al. (2024)
12	*G.hokouensis*	China: Fujian: Mt. Wuyi	SYS r001290	MW451647	OR902173	Lyu et al. (2021); Wang et al. (2024)
13	*G.japonicus*	China: Fujian: Mt. Wuyi	SYS r000672	MW451628	OR902176	Lyu et al. (2021); Wang et al. (2024)
14	*G.japonicus*	China: Jiangxi: Lushan	SYS r001317	MW451649	OR902177	Lyu et al. (2021); Wang et al. (2024)
15	*G.jinjiangensis*	China: Yunnan: Deqin	CIB 5334220088	—	MT449431	Hou et al. (2021)
16	*G.kaiyai*	China: Henan: Xinyang: Xinxian	AHUXXBH01	OQ780318	—	Zhang et al. (2023)
17	*G.kaiyai*	China: Henan: Xinyang: Xinxian	AHUXXBH02	OQ780319	—	Zhang et al. (2023)
18	*G.khunkhamensis*	Laos: Khammouane	VNUF R.2021.23	—	OL416111	Sitthivong et al. (2021)
19	*G.kwangsiensis*	China: Guangxi: Wuming	SYS r001194	MW451641	OR902174	Lyu et al. (2021); Wang et al. (2024)
20	*G.kwangsiensis*	China: Guangxi: Wuming	SYS r001195	MW451642	OR902175	Lyu et al. (2021); Wang et al. (2024)
21	***G.liboensis***	**China: Guizhou: Libo: Maolan**	**SYS r002876**	**PV652777**	**PV657381**	**This study**
22	***G.liboensis***	**China: Guizhou: Libo: Maolan**	**SYS r002877**	**PV652778**	**PV657382**	**This study**
23	*G.melli*	China: Guangdong: Dongyuan	SYS r001742	MW451661	OR902169	Lyu et al. (2021); Wang et al. (2024)
24	*G.nadenensis*	Laos: Khammouane	ZFMK 98741	—	KY421618	Luu et al. (2017)
25	*G.palmatus*	China: Guangdong: Mt.Dinghu	SYS r002797	OR903156	OR902179	Wang et al. (2024)
26	*G.paucituberculatus*	China: Guangxi: Baise: Tianyang	SYS r002806	OR903154	OR902163	Wang et al. (2024)
27	*G.paucituberculatus*	China: Guangxi: Baise: Tianyang	SYS r002807	OR903155	OR902164	Wang et al. (2024)
28	*G.scabridus*	China: Sichuan: Yanbian	CIB YN201909199	PQ255992	MT449429	Hou et al. (2021); Ma et al. (2024)
29	*G.scientiadventura*	Vietnam: Quang Binh	IEBR A.2014.7	—	KP205392	Luu et al. (2014)
30	*G.sengchanthavongi*	Laos: Khammouane	VFU R2014.14	—	KT266816	Luu et al. (2015)
31	*G.similignum*	China: Hainan: Mt. Wuzhi	SYS r001597	MW451658	OR902185	Lyu et al. (2021); Wang et al. (2024)
32	*G.subpalmatus*	China: Zhejiang: Fenghua	SYS r001762	MW451662	OR902167	Lyu et al. (2021); Wang et al. (2024)
33	*G.swinhonis*	China: Hebei: Zunhua	SYS r001814	MW451666	OR902171	Lyu et al. (2021); Wang et al. (2024)
34	*G.thakhekensis*	Laos: Khammouane: Thakhek	IEBR A.2014.6	—	KP205396	Luu et al. (2014)
35	*G.truongi*	Vietnam: Khanh Hoa	IEBR A.2011.1	—	KP205398	Luu et al. (2014)
36	*G.gecko*	China: Guangxi: Nanning	N/A	AY282753	AY282753	Zhou et al. (2006)
37	*G.reevesii*	China: Guangdong: Mt. Yinping	SYS r000796	MW451630	OR902187	Lyu et al. (2021); Wang et al. (2024)

Genomic DNA was extracted from liver tissue using a DNA extraction kit (Tiangen Biotech Co., Ltd, Beijing). Two fragments of the mitochondrial genes that encode partial 16S ribosomal RNA gene (16S) and partial NADH dehydrogenase subunit 2 gene (ND2) were amplified. Primers used for two genes are obtained from Simon et al. (1994) and Jonniaux and Kumazawa (2008). PCR amplifications were processed with the cycling conditions that initial denaturing step at 95 °C for 4 min and 5 min (16S and ND2, respectively), 35 cycles of denaturing at 95 °C for 40 s, annealing at 53 °C for 34 s (16S) and 55 °C for 40 s (ND2), extending at 72 °C for 60 s, and a final extending step at 72 °C for 10 min. PCR products were purified with spin columns and then sequenced with a forward primer using BigDye Terminator Cycle Sequencing Kit (Applied Biosystems, Waltham, MA, USA). Sequencing was performed on an ABI Prism 3730 automated DNA sequencer by Wuhan Tianyi Huiyuan Bioscience and Technology Inc.

DNA sequences were aligned by the MUSCLE algorithm with default parameters (Edgar 2004). PartitionFinder2 was used to determine the best partitioning scheme (Lanfear et al. 2017)and jModelTest v. 2.1.2 was used to determine the best fitting nucleotide substitution models (Darriba et al. 2012), resulting in the partitions by gene and ND2 was further partitioned by codon position and the best fit models for all partitions as GTR + I + G. Sequenced data were analyzed using Bayesian Inference (BI) in MrBayes 3.2.4 (Ronquist et al. 2012) and Maximum Likelihood (ML) in RaxmlGUI 1.3 (Silvestro and Michalak 2012). Two independent runs were conducted in the BI analysis with 2,000,000 generations each and sampled every 1000 generations with the first 25% of samples discarded as burn-in, resulting in a potential scale reduction factor (PSRF) of < 0.005. In the ML analysis, a bootstrap consensus tree inferred from 1000 replicates was generated. MEGA11 was used to calculate uncorrected pairwise sequence divergence for 16S and ND2 among and within species using the complete deletion option which removes missing data and gaps (Tamura et al. 2021).

## ﻿Results

### ﻿Morphological analyses

**Figure 2.**
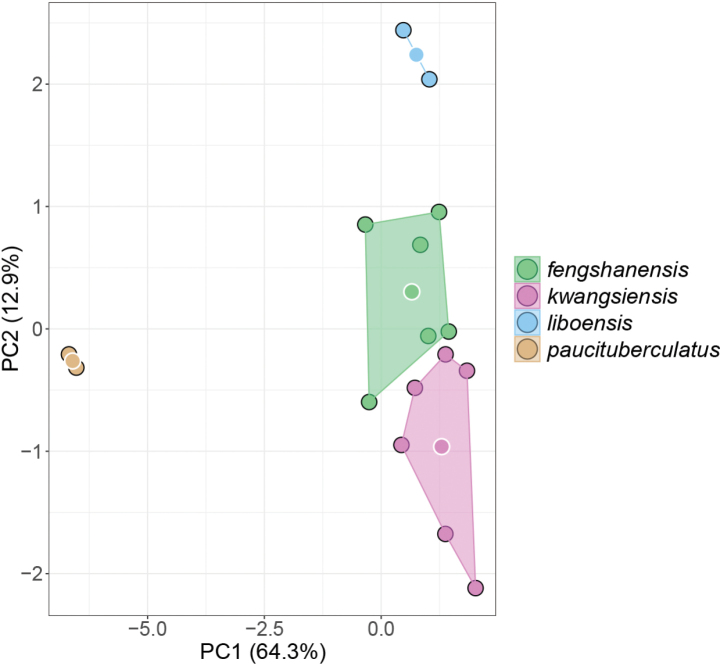
Scatter plot of PC1 and PC2 of Principal Component Analysis based on the morphometric measurements.

**Figure 3.**
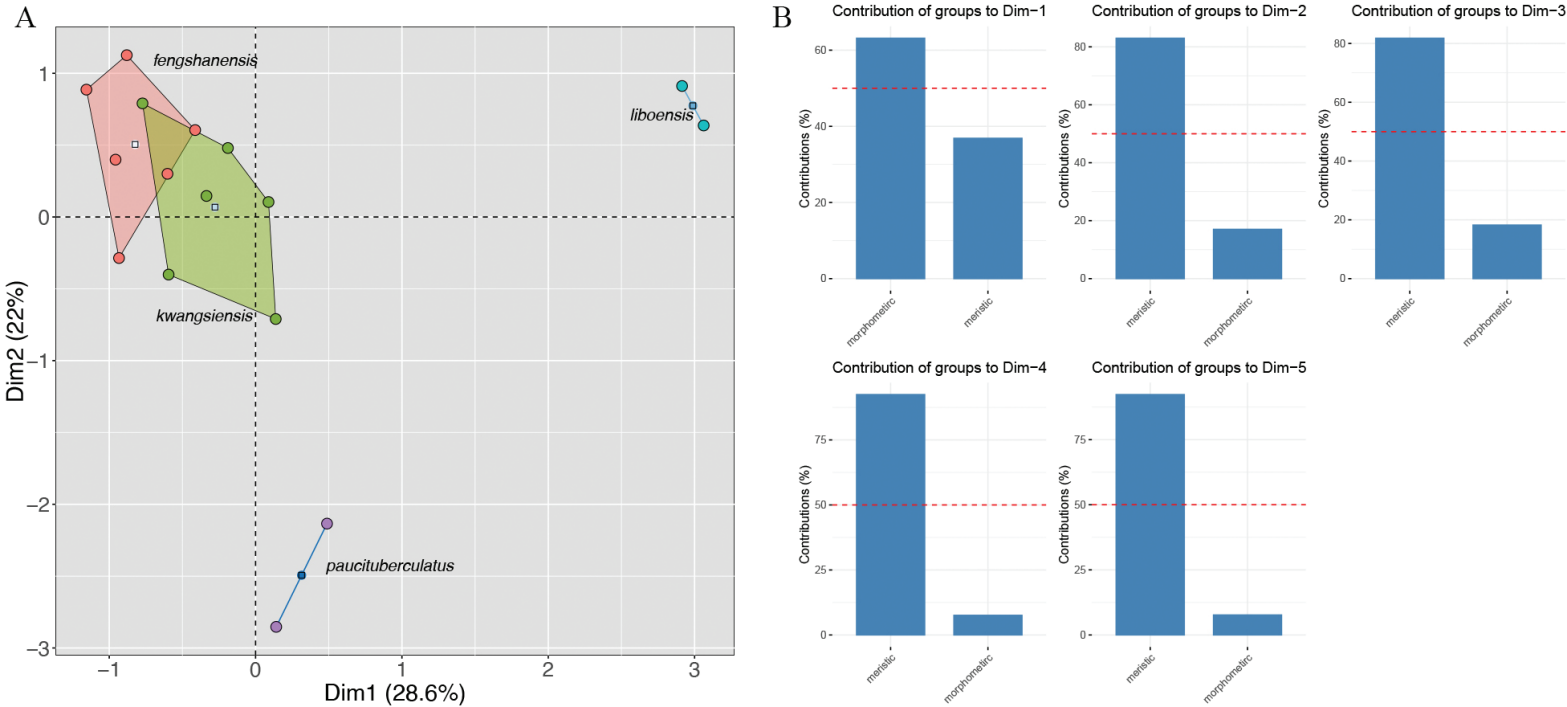
**A.**MFA of *Gekkofengshanensis* sp. nov., *G.kwangsiensis*, *G.liboensis*, and *G.paucituberculatus*; **B.** Percent contributions of each data type to the inertia of dimensions 1–4 of the MFA. Percentage values on the bar graphs are the amounts of inertia for their respective dimensions.

The results of one-way ANOVA of morphometrics (Table 3) show that the *Japonigekko* populations from Fengshan County is significantly different from *Gekkokwangsiensis*, especially in the characteristics of HL, ED, SNT, MW, and ML. In PCA analysis, the extracted components PC1, PC2, PC3, and PC4 eigenvectors account for 64.25%, 12.92%, 8.62%, and 6.10% of the variance, respectively, or 91.89% cumulatively. As illustrated in the scatter plots of PC1 and PC2 (Fig. 2), samples from Fengshan County cluster together and do not overlap with other species. However, the PERMANOVA analyses indicated that the centroid locations of the Fengshan population in the PCA are statistically not significantly different from *G.kwangsiensis* (Table 4). In the MFA, the Fengshan population and *G.kwangsiensis* showed slight overlap in morphospace (Fig. 3A) but were significantly different based on PERMANOVA (adjusted *p*-vaule < 0.05, Table 4). Dimension 1, which accounted for 28.6% of the total variation, was primarily influenced by morphometric traits and contributed most to the separation of species along the primary axis (Fig. 3B). In contrast, Dimensions 2–4 were dominated by meristic data, highlighting their role in further resolving interspecific differences beyond the major morphometric patterns.

**Table 3.** Morphometric comparisons based on the morphometric measurements of *Gekkofengshanensis* sp. nov. (*n* = 6) and *G.kwangsiensis* (*n* = 6). * *p*-values < 0.05, ** *p*-values < 0.01, *** *p*-values < 0.001.

	*G.fengshanensis* sp. nov.	*G.kwangsiensis*	F values	*p*-values
SVL	54.1–79.9	53.8–69.7	0.66876	0.432537
	65.95 ± 9.45	61.92 ± 6.49		
AG	24.7–34.2	23.4–32.6	0.366798	0.558249
	29.78 ± 3.86	27.62 ± 3.28		
HL	14.1–20.3	14.2–19.1	18.57999	0.001536**
	16.90 ± 2.37	16.77 ± 1.79		
HW	11.6–16.0	11.2–14.4	1.963985	0.191352
	14.00 ± 1.77	12.90 ± 1.28		
HH	5.5–9.1	6.0–7.7	2.043348	0.183361
	7.12 ± 1.35	7.03 ± 0.81		
ED	3.8–5.7	3.5–4.7	11.42863	0.006995**
	4.73 ± 0.65	4.22 ± 0.49		
SNT	6.0–8.8	6.3–8.4	8.377919	0.015984*
	7.55 ± 1.06	7.47 ± 0.85		
RW	2.5–3.2	2.3–3.0	3.082883	0.109644
	2.73 ± 0.27	2.76 ± 0.30		
RH	1.1–1.6	1.1–1.6	2.014796	0.186184
	1.37 ± 0.18	1.35 ± 0.16		
MW	1.6–2.3	1.9–2.4	8.324792	0.016238*
	2.02 ± 0.29	2.20 ± 0.20		
ML	1.0–1.4	1.3–1.7	27.29398	0.000387***
	1.15 ± 0.14	1.43 ± 0.18		

**Table 4.** PERMANOVA summary statistics for the centroid placement between all species pairs from the PCA and MFA analyses. Bold fonts denote insignificant adjusted *p*-values.

Species pairs	F. Model	R2	*p*-value	adjusted *p*-value
PCA statistics				
*G.fengshanensis* sp. nov. vs *kwangsiensis*	1.9464875	0.162933875	0.133037339	0.798224036
*G.fengshanensis* sp. nov. vs *liboensis*	0.191965061	0.031002284	0.892857143	1
*G.fengshanensis* sp. nov. vs *paucituberculatus*	10.68575178	0.640411767	0.035714286	0.214285714
*G.kwangsiensis* vs *liboensis*	1.418195136	0.191177923	0.392857143	1
*G.kwangsiensis* vs *paucituberculatus*	17.91796582	0.749142546	0.035714286	0.214285714
*G.liboensis* vs *paucituberculatus*	3.879400985	0.659829291	0.333333333	1
MFA statistics				
*G.fengshanensis* sp. nov. vs *kwangsiensis*	5.722068474	0.363951377	0.001939961	**0.011639767**
*G.fengshanensis* sp. nov. vs *liboensis*	14.22442629	0.703329038	0.035714286	0.214285714
*G.fengshanensis* sp. nov. vs *paucituberculatus*	9.340062225	0.608867297	0.035714286	0.214285714
*G.kwangsiensis* vs *paucituberculatus*	8.838729531	0.595652715	0.035714286	0.214285714
*G.kwangsiensis* vs *liboensis*	13.81080264	0.697134936	0.035714286	0.214285714
*G.liboensis* vs *paucituberculatus*	17.9312	0.899655	0.333333	1

### ﻿Phylogenetic analyses

**Figure 4.**
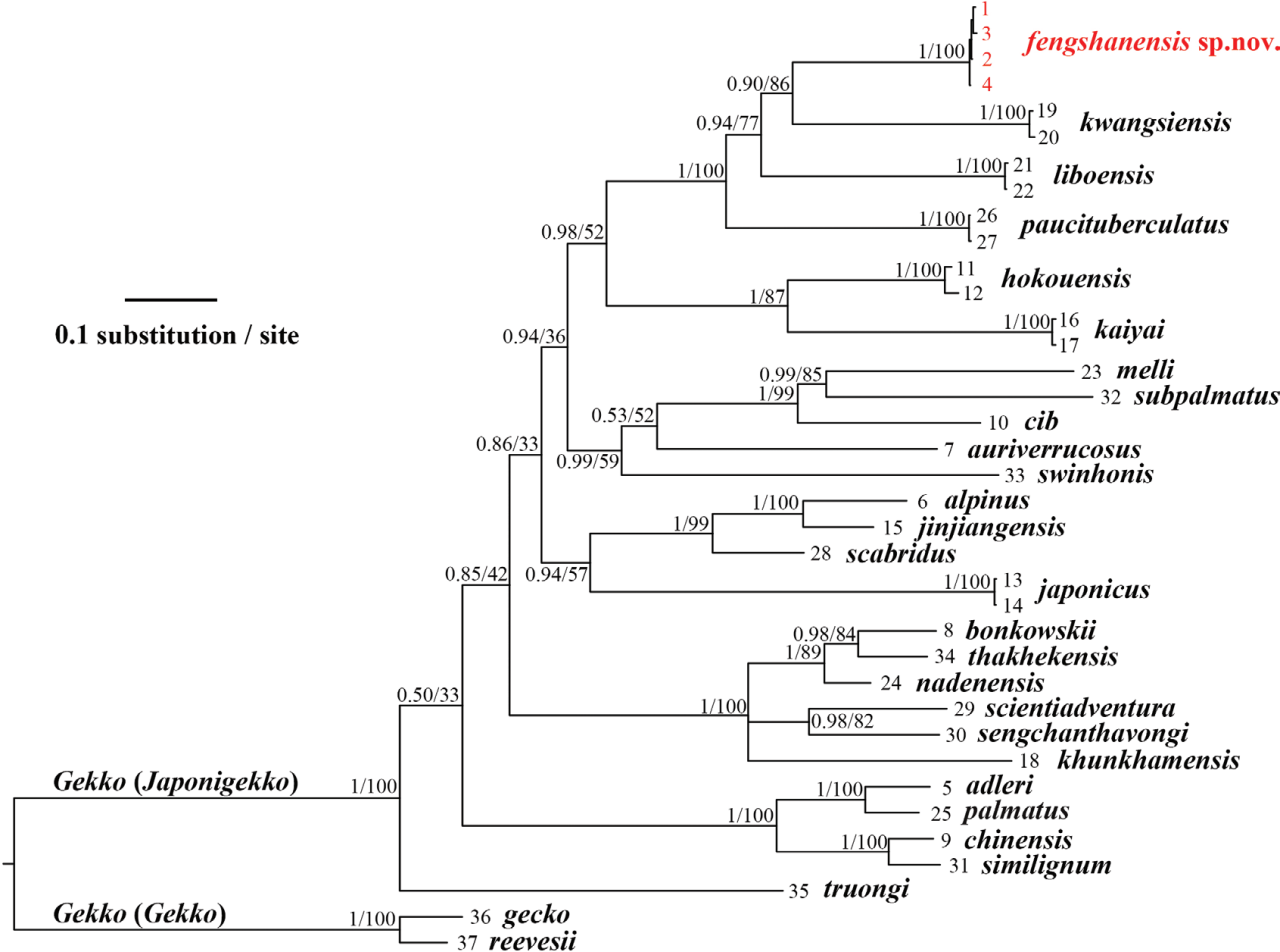
Bayesian inference tree inferred from16S and ND2 genes. Numbers before slash indicate Bayesian posterior probabilities (BPP) and numbers after slash are bootstrap support (BS).

The aligned dataset contained a total of 1578 nucleotide base pairs (bp), with 559 bp for 16S and 1015 bp for ND2. The BI and ML analyses resulted in essentially identical topologies (BI topology with ML bootstrap values in Fig. 4). The mean uncorrected *p* distances based on 16S and ND2 used in this study are given in Tables 5, 6. In both analyses, the newly collected samples from Fengshan County consistently formed a strongly supported (BPP = 1.00; BS = 100) monophyletic lineage within the subgenus Japonigekko. Moreover, the phylogenetic tree revealed that this lineage is sister to *Gekkokwangsiensis* with moderate support (BPP = 0.90; BS = 80), and together they are sister to *G.liboensis*. Finally, the three aforementioned taxa, together with *G.paucituberculatus*, formed a strongly supported endemic clade that exclusively inhabits the South China Karst. Based on uncorrected *p* distances, the new samples exhibited 8.20% (*G.liboensis*) to 10.32% (*G.paucituberculatus*) divergence in the 16S and 14.11% (*G.paucituberculatus*) to 16.36% (*G.kwangsiensis*) divergence in the ND2 gene compared to the other three species.

**Table 5.** Uncorrected *p* distances (%) of the 16S gene amongst species of *Japonigekko* used in this study.

	Species	1	2	3	4	5	6	7	8	9	10	11	12	13	14	15	16
1	*G.fengshanensis* sp. nov.	0.00															
2	*G.adleri*	15.08	—														
3	*G.alpinus*	12.96	14.29	—													
4	*G.chinensis*	15.08	3.70	12.43	—												
5	*G.cib*	12.70	14.81	9.52	12.96	—											
6	*G.hokouensis*	13.10	15.21	13.10	13.62	12.57	0.26										
7	*G.japonicus*	13.49	15.08	10.85	14.02	12.70	12.83	0.00									
8	*G.kaiyai*	12.96	14.02	13.76	12.70	11.90	6.75	13.23	0.00								
9	*G.kwangsiensis*	8.73	14.81	12.83	13.36	12.57	12.17	13.89	11.38	0.53							
10	*G.liboensis*	8.20	13.76	11.64	12.43	11.64	12.83	11.90	11.90	9.52	0.00						
11	*G.melli*	12.17	12.96	9.26	12.17	5.29	12.30	12.70	11.64	12.83	10.85	—					
12	*G.palmatus*	16.14	2.65	13.23	3.44	14.02	14.15	14.81	13.23	14.02	13.76	12.70	—				
13	*G.paucituberculatus*	10.32	15.34	11.11	13.76	12.17	11.77	14.55	12.17	10.32	7.41	12.96	14.29	0.00			
14	*G.scabridus*	12.17	13.49	5.56	12.70	10.05	10.98	10.85	11.90	13.36	11.64	10.85	12.70	10.32	—		
15	*G.similignum*	16.40	3.97	13.23	1.32	14.29	13.89	13.76	12.96	14.68	12.96	12.96	3.70	15.08	12.96	—	
16	*G.subpalmatus*	14.55	15.08	10.32	14.02	6.61	14.15	14.02	12.43	12.83	13.76	6.08	14.29	15.34	12.43	14.81	—
17	*G.swinhonis*	15.61	16.67	14.02	15.61	11.64	14.15	14.29	13.76	15.74	13.49	12.17	16.14	15.34	14.55	15.87	13.23

**Table 6.** Uncorrected *p* distances (%) of the ND2 gene amongst species of *Japonigekko* used in this study.0

	Species	1	2	3	4	5	6	7	8	9	10	11	12	13	14	15	16	17	18	19	20	21	22	23	24
1	*G.fengshanensis* sp. nov.	0.00																							
2	*G.adleri*	21.27	—																						
3	*G.alpinus*	19.43	21.27	—																					
4	*G.auriverrucosus*	20.04	24.13	20.86	—																				
5	*G.bonkowskii*	19.63	24.54	19.22	21.88	—																			
6	*G.chinensis*	22.09	14.52	21.47	25.15	20.86	—																		
7	*G.cib*	20.86	23.72	22.29	19.22	19.22	23.31	—																	
8	*G.hokouensis*	19.73	21.88	18.51	20.14	19.63	22.09	22.09	1.84																
9	*G.japonicus*	21.06	23.52	19.22	19.02	20.45	22.29	23.11	21.06	0.00															
10	*G.jinjiangensis*	18.40	21.27	8.38	21.06	19.22	22.09	21.27	20.35	18.40	—														
11	*G.khunkhamensis*	22.29	25.97	21.47	24.34	15.34	24.34	21.06	24.13	22.70	21.27	—													
12	*G.kwangsiensis*	16.36	21.06	21.37	21.68	21.37	21.47	18.61	20.86	22.29	19.33	21.57	0.41												
13	*G.liboensis*	15.44	21.98	20.45	20.35	19.12	22.80	20.76	16.97	21.57	19.84	21.57	17.28	0.20											
14	*G.melli*	23.11	20.86	20.86	22.29	22.09	22.29	17.59	21.27	21.68	20.86	23.52	21.68	21.17	—										
15	*G.nadenensis*	20.65	23.52	19.22	21.06	6.95	21.27	20.86	22.29	21.27	18.61	13.91	20.55	18.51	21.47	—									
16	*G.palmatus*	22.29	6.54	22.09	23.93	23.31	15.54	24.34	21.47	22.90	21.27	26.18	21.27	21.78	23.72	23.31	—								
17	*G.paucituberculatus*	14.11	22.09	17.59	18.61	18.81	22.70	19.02	18.92	19.84	18.20	21.68	15.54	15.85	21.27	18.61	21.27	0.00							
18	*G.scabridus*	16.16	18.61	11.86	19.43	19.84	20.45	21.27	17.28	17.79	10.84	21.88	19.84	17.48	20.45	19.43	19.63	18.00	—						
19	*G.scientiadventura*	19.63	24.34	18.81	21.06	13.70	22.70	21.47	21.68	22.09	18.61	14.72	20.86	20.14	22.09	13.91	23.52	19.02	17.79	—					
20	*G.sengchanthavongi*	20.04	23.93	20.45	20.65	13.91	21.68	21.68	22.90	21.68	19.84	15.75	20.86	19.73	22.49	12.07	23.72	19.22	18.81	10.43	—				
21	*G.similignum*	22.49	14.72	22.49	26.58	22.49	4.09	24.74	22.70	22.90	22.09	24.74	21.68	22.80	23.11	22.49	15.34	22.49	20.45	22.90	22.70	—			
22	*G.subpalmatus*	22.49	23.72	21.27	20.04	20.86	22.90	17.38	20.76	21.68	21.27	22.70	22.09	19.73	15.13	19.43	25.15	20.45	20.45	21.68	21.27	23.93	—		
23	*G.swinhonis*	20.86	22.70	21.47	18.81	21.68	22.90	21.27	19.94	20.86	21.06	23.52	19.84	20.14	20.65	21.68	22.90	21.47	20.04	23.11	21.88	22.29	20.25	—	
24	*G.thakhekensis*	19.63	21.68	19.43	21.06	7.16	19.63	20.45	20.96	20.04	19.63	15.95	20.35	18.51	21.06	6.95	22.70	17.79	19.02	13.09	12.88	20.45	20.45	23.72	—
25	*G.truongi*	23.31	19.84	20.86	24.54	22.09	19.84	21.68	21.98	22.29	21.06	22.29	22.19	21.37	20.86	22.29	21.47	20.86	17.79	22.09	21.88	20.45	21.47	23.72	20.45

In summary, when integrated with the morphological data, the phylogenetic results provide compelling evidence that the observed genetic differences of the new population represent a separate evolutionary lineage. Consequently, the data robustly support the recognition of this taxon as a new species of the subgenus Japonigekko.

### ﻿Taxonomic account

#### ﻿ Gekko fengshanensis sp. nov.

Taxon classificationAnimaliaSquamataGekkonidae

7054A24D-C9F1-5054-9B48-37530F2E6F29

https://zoobank.org/755C0A3B-57DC-4DE8-9B00-141BA36D54C4

Figs 5 , 6

##### Type material.

**Figure 5.**
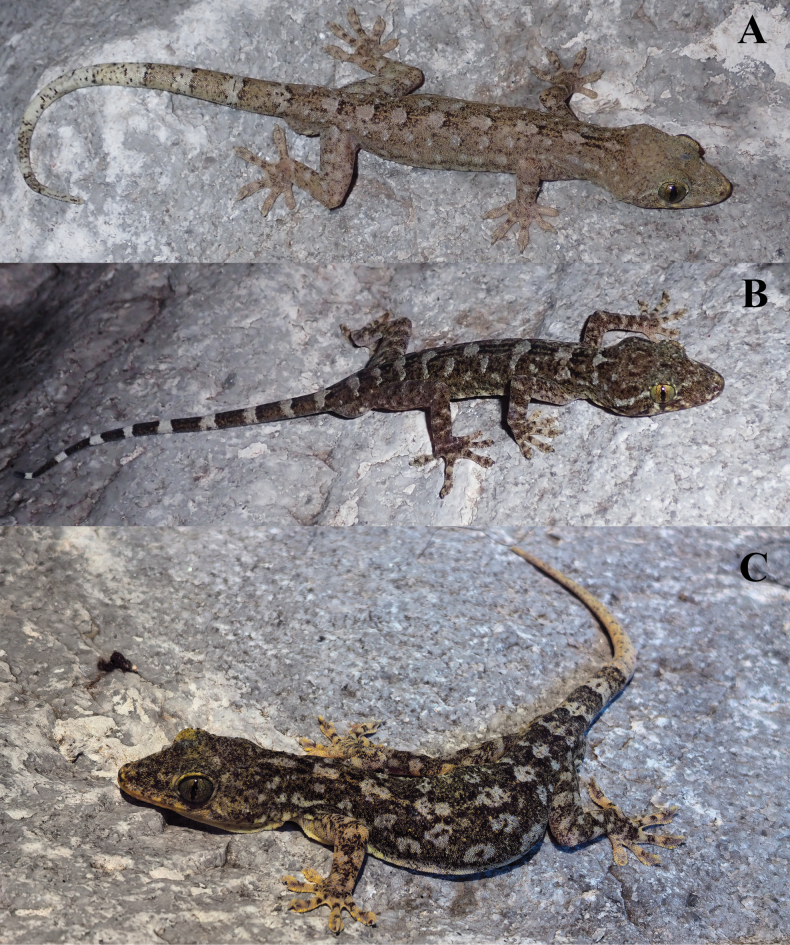
Type specimens of *Gekkofengshanensis* sp. nov. in life. **A.** Holotype NHMG 202408004, adult male; **B.** Paratype NHMG 202408005, adult male; **C.** Paratype NHMG 202408007, adult male. Photos by ZH.

**Figure 6.**
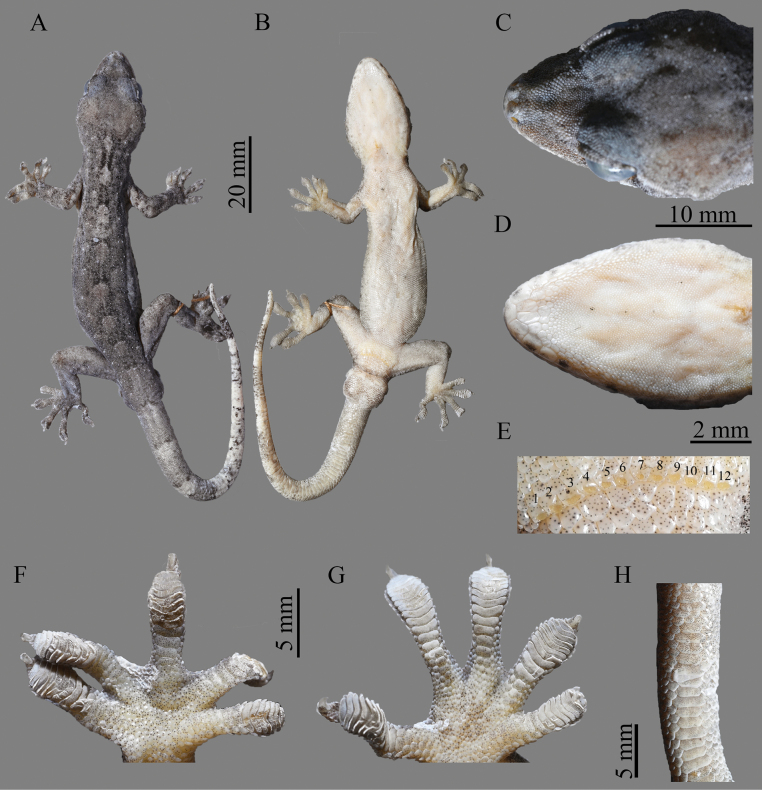
Morphological features of the adult male holotype NHMG 202408004 of *Gekkofengshanensis* sp. nov.; **A.** Dorsal view of body; **B.** Ventral view of body; **C.** Dorsal head; **D.** Ventral head; **E.** Precloacal pores; **F.** Left hand; **G.** Left foot; **H.** Ventral tail. Photos by HMS.

***Holotype*.** • NHMG 202408004 (Figs 5A, 6), adult male, from Fenghuang Village, Fengcheng Town, Fengshan County (24°31'59.25"N, 107°6'45.02"E; 680 m a.s.l.), Hechi City, Guangxi Zhuang Autonomous Region, China, collected on 5 August 2024 by Zhong Huang, Xiao-Wen Liao, Ben-Ze Huang and Yun-Ming Mo. ***Paratypes*.** • NHMG 202408005–007 (Fig. 5B, C), adult males, data identical to the holotype: NHMG 240713/SYS r002885, adult female, and NHMG 240714/SYS r002886, subadult male, from Xinglong Village, Fengcheng Town, Fengshan County (24°31'0.87"N, 107°4'23.97"E; 650 m a.s.l.), collected on 11 July 2024 by the same collector.

##### Diagnosis.

*Gekkofengshanensis* sp. nov. is assigned to the subgenus Japonigekko and distinguished from congeners by the following combination of characters: (1) moderate body size, SVL 60.0–79.9 mm in the adult male and SVL 62.2 in the adult female; (2) nares in contact with rostral, internasal absent; (3) enlarged postmentals two; (4) tubercles flattened, present from the region behind the eyes along the neck to the tail base, 8–11 rows at midbody ; (5) ventral scales between mental and cloacal slit 193–213; (6) midbody scale rows 149–161; (7) ventral scale rows 40–49; (8) subdigital lamellae on first fingers 11–13, on fourth fingers 12–16, on first toes 12–14, on fourth toes 13–15,and fingers and toes webbing weakly developed; (9) continuous precloacal pores 9–12 in males, absent in the female; (10) a single postcloacal tubercle on each side.

##### Description of holotype.

Adult male, moderate size, SVL 73.8 mm; head depressed (HH/HL 0.47), length longer than width (HL/HW 1.20), distinct from neck; snout rounded anteriorly, elongate (SNT/HL 0.42), larger than eye (SNT/ED 1.65); rostral regular rectangular, nearly twice as wide as high (RW/RH 1.93), and wider than the width mental (RW/MW 1.32); nares oval, bordered by rostral, first supralabial, supranasal, and two enlarged nasals posteriorly; internasals absent; preorbitals 18/19, preorbital region deeply concave; eye large (ED/HL 0.27), vertical pupil with crenulated margins; interorbital scales between anterior margins of eyes 27; ear opening elliptical, obliquely oriented, moderate in size (EOD/ED 0.33); mental pentagonal, wider than long (MW/ML 1.83); postmentals two, hexagonal and enlarged, twice as long as wide, touching mental and first infralabial on both sides and five gular scales posteriorly; supralabials 11/12; infralabials 11/10; tubercles present on the region behind the eyes, granular scales on anterodorsal region of head larger than those on posterior region.

Body slender, elongate (AG/SVL 0.46); dorsals smooth, round to oval, granular and juxtaposed; tubercles flattened, from postorbital region along the neck to tail base, nine rows at midbody, surrounded by 10 dorsal scales; ventrolateral fold present, without tubercles; ventrals distinctly larger than dorsals, smooth, imbricate, and largest in middle of belly; ventral scale rows at midbody 43; scale rows around midbody 149; ventral scales in a row between mental and cloacal slit 206; precloacal scales enlarged, but no enlarged scales on thighs; precloacal pores 12, in a continuous row across midline; postcloacal tubercle 1/1, large.

Fore- and hindlimbs well-developed; tubercles absent on dorsal surface of limbs; digits moderately dilated; II–IV fingers and toes clawed; claws depressed laterally, extending beyond terminal lamellae; webbing on fingers and toes weakly developed; subdigital lamellae undivided, manus for 12-11-13-15-12 (left) and 10-11-12-12-12 (right), pes for 13-11-14-13-11 (left) and 13-13-13-14-11 (right); relative length fingers and toes I < II < V < III < IV.

Original tail longer than body (TaL 87.3 mm, TaL/SVL 1.18); distinctly swollen at base; dorsal scales small, flat, smooth; caudal whorls distinct, 10 dorsal scale rows in the middle of the third one; subcaudals transversely enlarged.

##### Coloration of holotype.

In life, the dorsal regions of the head and body are light reddish-brown, with scattered white spots on the snout and posterior orbit. The iris is yellow-green with vermiform markings, and the pupil is dark brownish black. Seven irregularly shaped light patches are arranged along the ridge between the nape and the sacrum, with one or two rows of smaller spots parallelly arranged on each side. An intermittent light-colored vertebral line with black edges extends from the nape to the base of the tail. The anterior part of the tail exhibits a sharp contrast in color, which gradually fades and blends towards the posterior end. The ventral surface is lightly flesh-colored. In preservative, the dorsal ground color of the head, body, and limbs turns greyish black, while the ventral surface fades to greyish-white.

##### Morphological variation.

**Figure 7.**
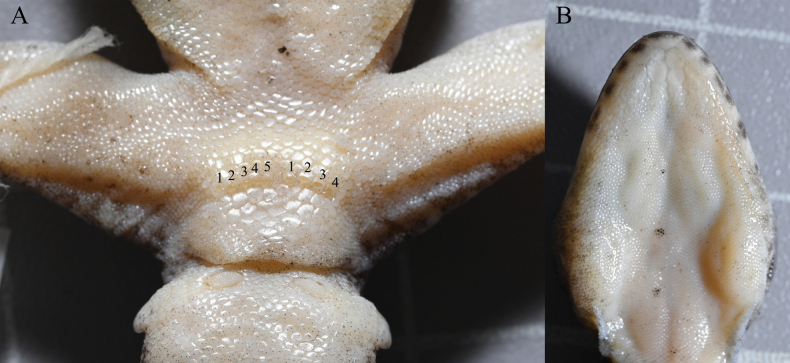
Morphological variation of *Gekkofengshanensis* sp. nov. **A.** Paratype NHMG 202408007, note the discontinuous precloacal pores; **B.** Paratype NHMG 202408007, note the postmentals. Photos by HMS.

Measurements and scale counts of six individuals are shown in Table 7. Precloacal pores are absent in the female. In males, the postcloacal tubercle is significantly larger than in the female. Except for those with a broken or regenerated tail, all paratypes have tails with a clear distinction between light and dark areas. NHMG 202408005 has a dark-brown “()”-shaped marking on the occipital region, and some of the dorsal patches combine with the spots on both sides to form a slightly larger, irregular patch (Fig. 5B). The light spots on the body of NHMG 202408007 are almost uniform in size, hollow, and resemble leopard spots (Fig. 5C). In terms of meristic traits, the precloacal pores of NHMG 202408007 are discontinuous, with four on the left side and five on the right side (Fig. 7A). The postmentals of NHMG 202408005 are slightly enlarged, and even smaller than the scales immediately following them (Fig. 7B).

**Table 7.** Measurements (in mm), body proportions, and scalation features of the type series of *Gekkofengshanensis* sp. nov. See Materials and Methods section for abbreviations. “*” regenerated tail; “—” unavailable data. Bilateral scale counts are given as left/right.

	Holotype	Paratypes				
Voucher Number	NHMG 202408004	NHMG 202408005	NHMG 202408006	NHMG 202408007	NHMG 240713/SYS r002885	NHMG 240714/SYS r002886
Sex	Male	Male	Male	Male	Female	Subadult male
SVL	73.8	60.0	65.7	79.9	62.2	54.1
TaL	87.3	67.1	—	74.3*	75.7	—
AG	34.1	26.9	30.4	34.2	28.4	24.7
HL	19.2	15.7	16.5	20.3	15.6	14.1
HW	16.0	12.9	14.3	16	13.2	11.6
HH	9.1	6.5	6.6	8.4	6.6	5.5
SNT	8.6	7.1	7.8	8.8	7.0	6.0
ED	5.2	4.5	4.6	5.7	4.6	3.8
EOD	1.7	1.3	1.6	1.9	1.7	1.2
RH	1.5	1.3	1.4	1.6	1.3	1.1
RW	2.9	2.7	2.6	3.2	2.5	2.5
MW	2.2	2.2	2.1	2.3	1.7	1.6
ML	1.2	1.1	1.1	1.4	1.1	1.0
TaL/SVL	1.18	1.12	—	0.93	1.22	—
AG/SVL	0.46	0.45	0.46	0.43	0.46	0.46
HL/SVL	0.26	0.26	0.25	0.25	0.25	0.26
HL/HW	1.20	1.22	1.15	1.27	1.18	1.22
HH/HL	0.47	0.41	0.40	0.41	0.42	0.39
SNT/HL	0.45	0.45	0.47	0.43	0.45	0.43
SNT/ED	1.65	1.58	1.70	1.54	1.52	1.58
ED/HL	0.27	0.29	0.28	0.28	0.29	0.27
EOD/ED	0.33	0.29	0.35	0.33	0.37	0.32
RW/RH	1.93	2.08	1.86	2.00	1.92	2.27
RW/MW	1.32	1.23	1.24	1.39	1.47	1.56
MW/ML	1.83	2.00	1.91	1.64	1.55	1.60
N	3/3	3/3	3/3	3/3	3/3	3/3
I	0	0	0	0	0	0
SPL	11/12	12/12	13/12	9/11	11/11	11/11
IFL	11/10	11/10	11/11	10/10	11/10	13/11
IO	27	26	24	22	25	25
PO	18/19	17/17	15/16	16/16	15/16	18/18
PM	2	2	2	2	2	2
GP	5	3	6	6	4	5
DTR	9	8	11	8	11	10
GSDT	10	9	9	9	9	9
SMC	206	197	205	213	198	193
SR	149	158	153	157	161	156
V	43	49	40	44	44	48
LF1	12/10	12/12	13/12	12/13	12/12	11/11
LF4	12/12	12/12	14/15	13/12	15/14	16/16
LT1	13/13	12/12	12/13	14/12	14/14	13/14
LT4	14/13	13/13	13/13	14/14	14/15	13/13
PP	12	11	11	9	—	12
PAT	1/1	1/1	1/1	1/1	1/1	1/1
S3W	10	9	9	11	10	10

**Table 8.** Scalation features comparisons among *Gekkofengshanensis* sp. nov., *G.kwangsiensis*, *G.liboensis* and *G.paucituberculatus*; differences are marked in bold.

Species	*G.fengshanensis* sp. nov.	*G.kwangsiensis*	*G.liboensis*	*G.paucituberculatus*
	*n* = 6	*n* = 6	*n* = 2	*n* = 2
Max SVL (mm)	79.9	**69.7**	79.7	**85.9**
N	3	3	3	3
I	0	0 or 1	0	0
SPL	9–13	10–13	11	11
IFL	10–13	11–13	9–11	9–10
IO	22–27	**29–31**	**32–35**	**37**
PO	15–19	18–20	17–18	14–18
PM	2	2	2	2
GP	3–6	4–6	4–6	4–6
DTR	9–11	9–11	9–10	**4**
GSDT	9–10	8–10	9–10	8
SMC	197–213	185–208	**183–195**	**189–192**
SR	149–161	143–156	**131–140**	**136–142**
V	40–49	41–45	38–41	42–44
LF1	11–13	10–13	12–13	10–11
LF4	12–16	12–14	14–17	12–13
LT1	12–14	11–13	12–13	11
LT4	13–15	14–18	14–15	11–13
PP	9–12	9–10	9	12
PAT	1	1	1	1

##### Etymology.

The specific epithet *fengshanensis* refers to Fengshan County, the type locality in Guangxi Zhuang Autonomous Region, China. The common name “Fengshan gecko” (English) and formal Chinese name “凤山壁虎” (fèng shān bì hǔ) are proposed.

##### Comparisons.

The molecular analyses indicated that *Gekkofengshanensis* sp. nov. is sister to *G.kwangsiensis*, and together they form a clade with *G.liboensis* and *G.paucituberculatus*, to which it is also morphologically similar. Morphological comparisons and analyses revealed their differences (Tables 3, 8). The new species differs from *G.kwangsiensis* in mean values of HL, ED, SNT, MW, ML (Table 3) and by having fewer interorbitals (22–27 vs 29–31); differs from *G.liboensis* by having fewer interorbitals (22–27 vs 32–35), more scales in a line from mental to the front of cloacal slit (197–213 vs 183–195), and more scale rows at midbody (149–161 vs 131–140); differs from *G.paucituberculatus* by having smaller maximum SVL (79.9 vs 85.9), fewer interorbitals (22–27 vs 37), more dorsal tubercle rows at midbody (9–11 vs 4), more scales in a line from mental to the front of cloacal slit (197–213 vs 189–192), more scale rows at midbody (149–161 vs 136–142), and coloration pattern (irregular light patches with lateral spots vs dirty-white transverse bands between the nape and sacrum).

For the remaining congeners, the new species differs from the following 13 congeners by the presence of tubercles on dorsolateral trunk: the absence of tubercles in *Gekkoaaronbaueri*, *G.bonkowskii*, *G.cib*, *G.guishanicus*, *G.khunkhamensis*, *G.melli*, *G.nadenensis*, *G.scientiadventura*, *G.sengchanthavongi*, *G.subpalmatus*, *G.tawaensis*, *G.thakhekensis*, and *G.truongi*; differs from the following 13 congeners by having 9–12 precloacal pores in males: *Gekkoadleri* (17–21), *G.alpinus* (4–7), *G.canhi* (5), *G.chinensis* (17–27), *G.jinjiangensis* (4–5), *G.palmatus* (23–30), *G.shibatai* (0), *G.similignum* (17), *G.taibaiensis* (4–6), *G.vertebralis* (0), *G.vietnamensis* (0), *G.wenxianensis* (6–8) and *G.yakuensis* (6–8); differs from *G.kaiyai* by the absence of tubercles on limbs (vs present); differs from *G.hokouensis* by having more scale rows at midbody (149–161 vs 119–130); differs from *G.auriverrucosus* and *G.scabridus* by having fewer dorsal tubercle rows (8–11 vs 16–20 and 17–21, respectively); differs from *G.ichangensis*, *G.japonicus* and *G.swinhonis* by having a single postcloacal tubercles (vs 3, 2–4 and 2 or 3, respectively).

##### Distribution and ecology.

**Figure 8.**
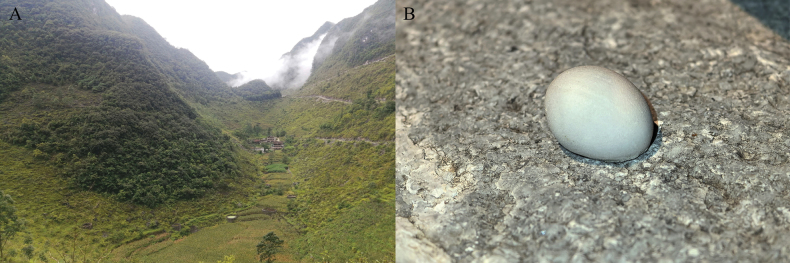
**A.** Habitats of *Gekkofengshanensis* sp. nov.; **B.** The egg of *Gekkofengshanensis* sp. nov. Photos by ZH.

Currently, *Gekkofengshanensis* sp. nov. is known only from Fengshan County, Hechi City, Guangxi Zhuang Autonomous Region, China. All six individuals were discovered at night on the walls of artificial buildings located near the karst forests (Fig. 8A). Other amphibian and reptile species co-occurring with it include *Kurixalushainanus* (Zhao, Wang, & Shi, 2005), *Pseudocalotesmicrolepis* (Boulenger, 1888), and *Sinomicruruspeinani* Liu, Yan, Hou, Wang, Nguyen, Murphy, Che, & Guo, 2020.

At the type locality, one milky-white, elliptical egg measuring ~1.5 cm in diameter was found in a crevice of a house (Fig. 8B). It is presumed to belong to this species, suggesting that the breeding season likely occurs in July and August.

##### Additional specimens examined.

*Gekkoliboensis* (*n* = 2): SYS r002876 (adult male) and SYS r002877 (subadult female), from Weng’ang Station, Maolan National Nature Reserve, Libo County, Guizhou Province, China, collected on 24 July 2024 by Hao-Tian Wang and Ying-Yong Wang.

*Gekkopaucituberculatus* (*n* = 2): SYS r002806 (adult male) and SYS r002807 (adult female), from Tianyang District, Baise City, Guangxi Zhuang Autonomous Region, China, collected on 3 August 2023 by Dan-Yang Zhou.

The GenBank accession numbers for the four specimens listed above can be found in Table 2, and the corresponding morphological data are provided in Suppl. material 1. These specimens are deposited in the Sun Yat-sen University (SYS), Guangzhou, China.

## ﻿Discussion

The discovery of *Gekkofengshanensis* sp. nov. represents a significant addition to the biodiversity of the South China Karst, bringing the total number of recognized *Gekko* species inhabiting this unique karst ecosystem to five. Molecular phylogenetic analyses revealed that these species (*Gekkofengshanensis* sp. nov., *G.kwangsiensis*, *G.liboensis*, and *G.paucituberculatus*), excluding *G.adleri*, form a monophyletic lineage, indicating their shared evolutionary origin within karst habitats. This finding strongly supports the hypothesis that the fragmented nature of karst landscapes (e.g., isolated peak clusters, depressions, and canyons) has limited or only historical gene flow among populations, driving allopatric speciation (Chung et al. 2014; Grismer et al. 2020, 2021). The three species — *G.fengshanensis* sp. nov. (mid- to high-elevation canyons), *G.kwangsiensis* (isolated Daming Mountain, Youjiang River basin), and *G.paucituberculatus* (low-elevation limestone in the Baise Basin) — occupy ecologically distinct habitats across their largely allopatric ranges. Their distributions are confined to the western side of the Hongshui River, which, together with the Longjiang River, forms a biogeographic barrier that isolates them from *G.liboensis* populations to the east (Fig. 1). Dispersal among these species is likely constrained by intervening non-karst terrain, with the dual-river system coinciding with species boundaries and potentially contributing to the reinforcement of genetic divergence. The karst region’s complex landforms have played a crucial role in shaping biodiversity by creating fragmented and unique microhabitats. These diverse landscapes have fostered a high degree of endemism and species richness (Clements et al. 2006; Grismer et al. 2021). Grismer et al. (2021) further confirmed that karst landscapes actively drive species diversification within *Cyrtodactylus*, rather than merely serving as “imperiled arks of biodiversity” that act as refugia for relic species. This pattern aligns with the diversification of *Gekko* species in the South China Karst, where geographic isolation and habitat specialization have been key drivers of speciation.

*Gekkoliboensis* was originally described by Zhou et al. (1982) based on a single female specimen from Libo County, Guizhou Province. However, its taxonomic validity has long been debated due to its extreme rarity, with only four specimens ever recorded (Zhao et al. 1999). Zhao and Adler (1993), Günther (1994), and Kluge (2001) considered it a junior synonym of *G.hokouensis*, whereas Kluge (1993), Bauer (1994), Matsui and Ota (1995), Zhao et al. (1999), and Rösler et al. (2011) recognized it as a valid species. Based on newly collected specimens from Libo, Guizhou (the type locality of *G.liboensis*), and Guangxi, Jono et al. (2015) employed morphometric methods to confirm the validity of *G.liboensis*, while also extending its known distribution to Guangxi. In the present study, we further validate its taxonomic status based on the molecular evidence of topotypes newly collected for this study. The *G.liboensis* specimens reported from Guangxi by Jono et al. (2015) were recorded in Nalao (24°25'N, 107°22'E), but both the locality name and its coordinates are imprecise. There are several villages named Nalao or with similar names in this vicinity (the “?” symbol in Fig. 1), specifically Nalang Village, Sanshi Town, Donglan County (24°24'35.95"N, 107°21'20.83"E); Nalao Village, Wuzuan Town, Donglan County (24°21'33.01"N, 107°16'49.26"E); and Nalao Village, Xishan Town, Bama Yao Autonomous County (24°14'13.12"N, 107°18'07.02"E). All these localities lie on the western side of the Hongshui River and are in proximity to the type locality of *G.fengshanensis* sp. nov. (~35 km). Given the striking morphological similarities between the two species, the specimens identified as *G.liboensis* by Jono et al. (2015) from Guangxi likely represent misidentifications of *G.fengshanensis* sp. nov. Consequently, the reported occurrence of *G.liboensis* in Guangxi remains questionable and necessitates further verification.

## Supplementary Material

XML Treatment for Gekko fengshanensis

## ﻿Additional information

### Use of AI

No use of AI was reported.

### Author ORCIDs

Zhong Huang https://orcid.org/0009-0001-7825-4778

Hao-Tian Wang https://orcid.org/0000-0002-2415-3234

Shuo Qi https://orcid.org/0000-0002-2924-6093

Han-Ming Song https://orcid.org/0009-0005-8244-1567

### Data availability

All of the data that support the findings of this study are available in the main text or Supplementary Information.

## Supplementary materials

Supplementary material 1 Raw morphological data used in PCA and MFA analyses Data type xlsx https://binary.pensoft.net/file/1374358This dataset is made available under the Open Database License (http://opendatacommons.org/licenses/odbl/1.0/). The Open Database License (ODbL) is a license agreement intended to allow users to freely share, modify, and use this Dataset while maintaining this same freedom for others, provided that the original source and author(s) are credited.Zhong Huang, Hao-Tian Wang, Shuo Qi, Han-Ming Song, Yong Huang, Ying-Yong Wang, Yun-Ming Mo
